# Supplementary material for: Signalling maps in cancer research: construction and data analysis
Source: Database (Oxford). 2018 Apr 9;2018:bay036. doi: 10.1093/database/bay036 (PMC5890450; doi:10.1093/database/bay036)
Supplement: Supplementary Data [file bay036_supp.docx]

**Supplementary materials**

| **Name** | **Website** | **Description** | **Reference** |
| --- | --- | --- | --- |
| **STRING** | http://string-db.org | Integrated protein-protein interaction daatabase | (1) |
| **BioGRID** | http://thebiogrid.org | Integrated protein-protein and genetic interaction daatabase | (2) |
| **MINT** | http://mint.bio.uniroma2.it/mint/Welcome.do | Carefully curated PPI resource | (3) |
| **PathwayCommons** | http://www.pathwaycommons.org | Biological pathways resource collected from public pathway databases | (4) |
| **TRANSPATH** | http://www.biobase-international.com | Database of mammalian signal transduction and metabolic pathways | (5) |
| **ConsensusPathDB** | http://consensuspathdb.org | Integrated resource of interaction networks and pathways | (6) |
| **Panther** | http://pantherdb.org | Collection of biological pathways and data visualization and analysis tools | (7) |
| **Spike** | http://www.cs.tau.ac.il/~spike | Collection of curated, peer reviewed pathways and data visualization tools | (8) |
| **WikiPathways** | http://www.wikipathways.org | Collection of community curated signalling pathways | (9) |
| **PID-NCI** | http://pid.nci.nih.gov | Curated collection of information about biomolecular interactions and signalling pathways |  |
| **KEGG Pathway** | http://www.genome.jp/kegg/pathway | Collection of manually drawn pathway maps visualization tool | (10) |
| **Reactome** | http://www.reactome.org | Collection of curated, peer reviewed pathways and data visualization/analysis tools | (11) |
| **ACSN** | http://acsn.curie.fr | Collection of curated, peer reviewed, interconnected cancer-related signaling networks and data visualization/analysis tools | (12) |
| **Parkinson’s disease map** | https://wwwen.uni.lu/lcsb/research/parkinson_s_disease_map | manually curated knowledge repository established to describe molecular mechanisms of PD | (13) |

**Supplementary Table 1: Pathways databases and network resources**

| **Biological diagram editors** | | | |
| --- | --- | --- | --- |
| **Name** | **Website** | **Description** | **Reference** |
| **CellDesigner** | http://www.celldesigner.org | Structured diagram editor for drawing gene-regulatory and biochemical networks | (14) |
| **SBGN-ED** | http://vanted.ipk-gatersleben.de/addons/sbgn-ed | VANTED add-on for create and edit three types of SBGN maps | (15) |
| **CellPublisher** | http://cellpublisher.gobics.de | KEGG database-associated tool for data visualization and analysis in the context of pathway maps | (16) |
| **Cytoscape / BiNoM** | http://www.cytoscape.org  http://binom.curie.fr | Software platform for maniputation of biological networks represented in standard systems biology formats | (17)  (18) |
| **Payao** | <http://payao.oist.jp:8080/payaologue/index.html> | Network curation tool for simultaneous map commenting using tag system | (19) |
| **NaviCell** | http://navicell.curie.fr | Web-based tool for heterogeneous data visualization and analysis in the context of signaling networks | (20)(21) |
| **yEd graph editor** | http://www.yworks.com/en/products/yfiles/yed/ | Application for generate high-quality diagrams construction | <http://link.springer.com/chapter/10.1007/978-3-642-18638-7_8> |
| **VisANT** | http://visant.bu.edu/ | Tool for visual analyses of metabolic networks in cells and ecosystems | (22) |
| **Pathway Map Creator** | http://lifesciences.thomsonreuters.com/m/pdf/PathwayMapCreator-cfs-en.pdf | Tool for editing and analysis of canonical pathways maps |  |
| **Tools for visualisation of high-throughput data in the context of signalling networks** | | | |
| **Name** | **Website** | **Description** | **Reference** |
| **ReactomeFiViz** | http://wiki.reactome.org/index.php/Reactome_FI_Cytoscape_Plugin | Cytoscape plugin for data integration into signaling networks | (23) |
| **iPAth** | http://pathways.embl.de | Web-based tool for data visualization in the context of pathway maps | (24) |
| **Medusa** | http://coot.embl.de/medusa | Tool for data visualization in the context of signaling network and network clustering | (25) |
| **NaviCell** | http://navicell.curie.fr | Web-based tool for heterogeneous data visualization and analysis in the context of signaling networks | (20)(21) |
| **NaviCom** | http://navicom.curie.fr | Web-based platform for generating interactive network based molecular portraits using high-throughput datasets. | (26) |
| **MINERVA** | http://r3lab.uni.lu/web/minerva-website | Webserver for visualization, exploration and management of molecular networks encoded in SBGN-compliant format | (27) |
| **KEGG Mapper** | http://www.kegg.jp/kegg/mapper | KEGG database-associated tool for data visualization and analysis in the context of pathway maps | (10) |

**Supplementary Table 2: Biological diagram editors, map navigation tools and high-throughput data visualization support**

**Supplementary Table 3: Method for studying molecular and genetic interactions**

| **Interaction** | **Method** | **Reference** |
| --- | --- | --- |
| **Ligand-receptor interactions** | Resonance energy transfer (FRET and BRET) | (28)(29) |
|  | Flow Cytometric Analysis | (30) |
| **Direct protein-protein interactions** | Co-immunoprecipitation (CoIP), | (31)(32) |
|  | NMR, X-ray crystallography, | (33) |
|  | GST-pull down assay | (34) |
|  | Tandem affinity purification | (31)(29) |
|  | Far Western blotting | (35) |
|  | Phage display | (32) |
|  | Mass-spectrometry | (36) |
|  | Two hybrid assays (yeast and mammalia) | (31) |
|  | Functional mutational analysis | (37) |
| **Direct protein-DNA interaction (transcription regulation and co-regulation)** | Chromatin immunoprecipitation (ChIP) | (38)(39) |
|  | DNA footprinting, | (38) |
|  | Electrophoretic mobility shift end supershift assays (EMSA) | (38) |
|  | Computational prediction of transcription factors binding sites | (40) |
| **MicroRNA binding** | Direct miRNA binding assay, | (41) |
|  | 3 'UTR reporter assay, | (41) |
|  | Computational miRNA target prediction | (41) |
| **Regulation of expression (mRNA and protein level)** | Reverse transcription polymerase chain reaction  (RT-PCR) | (42) |
|  | Reporter assays | (43) |
|  | RNase protection assay | (44) |
|  | Nothern blot | (42) |
|  | Western blot | (45) |
|  | Fluorescence-activated cell sorting (FACS) | (46) |
| **Genetic interactions** | Genetic knock-out, knock-down, knock in or  overexpression of effector molecules | (47)(48) |
|  | Synthetic interaction detection assays | (32)(49) |

**Supplementary Figure 1 Automatic text to diagram translation using BiNoM Reaction Format (BRF) language**


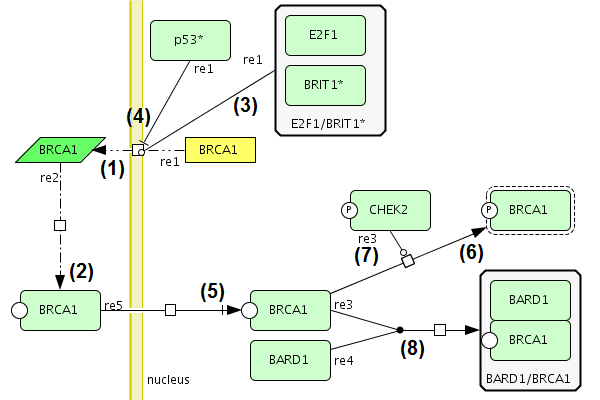


Representation of biochemical reactions from the following statements. Numbers correspond to the reactions in the diagram: *« BRCA1 transcription (1) and translation (2) is positively regulated by E2F1/BRIT1* complex (3) and inhibited by p53 (4). BRCA1 protein is transported into nucleus (5), where CHEK2 kinase activates it by specific phosphorylation (6) and (7). Additionally, BRCA1 forms a complex with BARD1 (8) and BRCA1 association with BARD1 is essential for the E3 ligase activity of BRCA1».* References correspondence: reactions 1,2,4 (50); reaction 3 (51); reaction 5 (52); reactions 6,7 (53); reaction 8 (54).

The set of corresponding statements in BiNoM Reaction Format (BRF) language were generated from the text and translated into the diagram:

BRCA1@cytoplasm -/> BRCA1@nucleus

BRCA1@nucleus+BARD1@nucleus -:> BARD1:BRCA1@nucleus

BRCA1-CHEK2|pho@nucleus -> BRCA1|pho|active@nucleus

rBRCA1@cytoplasm -.> BRCA1@cytoplasm

gBRCA1@nucleus -|p53*@nucleus -BRIT1*:E2F1@nucleus -..> rBRCA1@cytoplasm

**Supplementary Figure 2 Annotation page in the ACSN blog for BRCA1 protein**

**Access, documentation and tutorials**

**Maps construction:**

**CellDesigner introduction and tutorial**

http://celldesigner.org/documents.html

**SBGN**

http://www.sbgn.org/Main_Page

**BiNoM manual**

https://binom.curie.fr/docs/BiNoM_Manual_v2.pdf

**Preparing modular map in NaviCell format and converting to NaviCell web-based environment:**

https://github.com/sysbio-curie/NaviCell

**Data visualization and analysis:**

**NaviCell Web Service introduction, tutorial and case studies**

https://navicell.curie.fr/pages/nav_web_service.html

**NaviCell Web Service guide**

https://navicell.curie.fr/doc/ws/NaviCellWebServiceGuide.pdf

**Interactive demo on data visualization using NaviCell**

https://navicell.curie.fr/navicell/maps/cellcycle/master/index.php?demo=on

**NaviCom guide**

https://navicom.curie.fr/tutorial.pdf

**Maps access:**

**ACSN introduction, tutorial and case studies**

https://acsn.curie.fr/documentation.html

**NaviCell maps collection**

https://navicell.curie.fr/pages/maps.html

**References**

1. Franceschini, A., Szklarczyk, D., Frankild, S., et al. (2013) STRING v9.1: protein-protein interaction networks, with increased coverage and integration., *Nucleic Acids Res.*, **41**, D808-15.

2. Chatr-Aryamontri, A., Breitkreutz, B.-J., Oughtred, R., et al. (2015) The BioGRID interaction database: 2015 update., *Nucleic Acids Res.*, **43**, D470-8.

3. Licata, L., Briganti, L., Peluso, D., et al. (2012) MINT, the molecular interaction database: 2012 update., *Nucleic Acids Res.*, **40**, D857-61.

4. Cerami, E. G., Gross, B. E., Demir, E., et al. (2011) Pathway Commons, a web resource for biological pathway data., *Nucleic Acids Res.*, **39**, D685-90.

5. Schacherer, F., Choi, C., Götze, U., et al. (2001) The TRANSPATH signal transduction database: a knowledge base on signal transduction networks., *Bioinformatics*, **17**, 1053–7.

6. Kamburov, A., Stelzl, U., Lehrach, H., et al. (2013) The ConsensusPathDB interaction database: 2013 update., *Nucleic Acids Res.*, **41**, D793-800.

7. Mi, H., Lazareva-Ulitsky, B., Loo, R., et al. (2005) The PANTHER database of protein families, subfamilies, functions and pathways., *Nucleic Acids Res.*, **33**, D284-8.

8. Paz, A., Brownstein, Z., Ber, Y., et al. (2011) SPIKE: a database of highly curated human signaling pathways., *Nucleic Acids Res.*, **39**, D793-9.

9. Kelder, T., van Iersel, M. P., Hanspers, K., et al. (2012) WikiPathways: building research communities on biological pathways., *Nucleic Acids Res.*, **40**, D1301-7.

10. Kanehisa, M., Goto, S., Sato, Y., et al. (2012) KEGG for integration and interpretation of large-scale molecular data sets., *Nucleic Acids Res.*, **40**, D109-14.

11. Croft, D., Mundo, A. F., Haw, R., et al. (2014) The Reactome pathway knowledgebase., *Nucleic Acids Res.*, **42**, D472-7.

12. Kuperstein, I., Bonnet, E., Nguyen, H.-A., et al. (2015) Atlas of Cancer Signalling Network: a systems biology resource for integrative analysis of cancer data with Google Maps., *Oncogenesis*, **4**, e160.

13. Ghosh, S., Matsuoka, Y. and Kitano, H. (2010) Connecting the dots: role of standardization and technology sharing in biological simulation., *Drug Discov. Today*, **15**, 1024–31.

14. Kitano, H., Funahashi, A., Matsuoka, Y., et al. (2005) Using process diagrams for the graphical representation of biological networks, *Nat. Biotechnol.*, **23**, 961–966.

15. Czauderna, T., Klukas, C. and Schreiber, F. (2010) Editing, validating and translating of SBGN maps., *Bioinformatics*, **26**, 2340–1.

16. Flórez, L. A., Lammers, C. R., Michna, R., et al. (2010) CellPublisher: a web platform for the intuitive visualization and sharing of metabolic, signalling and regulatory pathways., *Bioinformatics*, **26**, 2997–9.

17. Zinovyev, A., Viara, E., Calzone, L., et al. (2008) BiNoM: a Cytoscape plugin for manipulating and analyzing biological networks., *Bioinformatics*, **24**, 876–7.

18. Bonnet, E., Calzone, L., Rovera, D., et al. (2013) BiNoM 2.0, a Cytoscape plugin for accessing and analyzing pathways using standard systems biology formats., *BMC Syst. Biol.*, **7**, 18.

19. Matsuoka, Y., Ghosh, S., Kikuchi, N., et al. (2010) Payao: a community platform for SBML pathway model curation., *Bioinformatics*, **26**, 1381–3.

20. Kuperstein, I., Cohen, D. P. A., Pook, S., et al. (2013) NaviCell: a web-based environment for navigation, curation and maintenance of large molecular interaction maps., *BMC Syst. Biol.*, **7**, 100.

21. Bonnet, E., Viara, E., Kuperstein, I., et al. (2015) NaviCell Web Service for network-based data visualization., *Nucleic Acids Res.*

22. Hu, Z., Mellor, J., Wu, J., et al. (2004) VisANT: an online visualization and analysis tool for biological interaction data., *BMC Bioinformatics*, **5**, 17.

23. Wu, G., Dawson, E., Duong, A., et al. (2014) ReactomeFIViz: a Cytoscape app for pathway and network-based data analysis., *F1000Research*, **3**, 146.

24. Yamada, T., Letunic, I., Okuda, S., et al. (2011) iPath2.0: interactive pathway explorer., *Nucleic Acids Res.*, **39**, W412-5.

25. Pavlopoulos, G. A., Hooper, S. D., Sifrim, A., et al. (2011) Medusa: A tool for exploring and clustering biological networks., *BMC Res. Notes*, **4**, 384.

26. Dorel, M., Viara, E., Barillot, E., et al. (2017) NaviCom: a web application to create interactive molecular network portraits using multi-level omics data, *Database*, **2017**.

27. Gawron, P., Ostaszewski, M., Satagopam, V., et al. (2016) MINERVA-a platform for visualization and curation of molecular interaction networks., *NPJ Syst. Biol. Appl.*, **2**, 16020.

28. Day, R. N. and Davidson, M. W. (2012) Fluorescent proteins for FRET microscopy: monitoring protein interactions in living cells., *Bioessays*, **34**, 341–50.

29. Piehler, J. (2005) New methodologies for measuring protein interactions in vivo and in vitro, *Curr. Opin. Struct. Biol.*, **15**, 4–14.

30. Sklar, L. a, Edwards, B. S., Graves, S. W., et al. (2002) Flow cytometric analysis of ligand-receptor interactions and molecular assemblies., *Annu. Rev. Biophys. Biomol. Struct.*, **31**, 97–119.

31. Berggård, T., Linse, S. and James, P. (2007) Methods for the detection and analysis of protein-protein interactions, *Proteomics*, **7**, 2833–2842.

32. Phizicky, E. M. and Fields, S. (1995) Protein-protein interactions: methods for detection and analysis., *Microbiol. Rev.*, **59**, 94–123.

33. Jubb, H., Higueruelo, A. P., Winter, A., et al. (2012) Structural biology and drug discovery for protein-protein interactions, *Trends Pharmacol. Sci.*, **33**, 241–248.

34. Sambrook, J. and Russell, D. W. (2006) Detection of Protein-Protein Interactions Using the GST Fusion Protein Pulldown Technique., *CSH Protoc.*, **2006**.

35. Wu, Y., Li, Q. and Chen, X.-Z. (2007) Detecting protein-protein interactions by Far western blotting., *Nat. Protoc.*, **2**, 3278–84.

36. Aebersold, R. and Mann, M. (2003) Mass spectrometry-based proteomics., *Nature*, **422**, 198–207.

37. Bogan, A. A. and Thorn, K. S. (1998) 1-s2.0-S0022283698918435-main.

38. Dey, B., Thukral, S., Krishnan, S., et al. (2012) DNA-protein interactions: Methods for detection and analysis, *Mol. Cell. Biochem.*, **365**, 279–299.

39. Stormo, G. D. and Zhao, Y. (2010) Determining the specificity of protein-DNA interactions., *Nat. Rev. Genet.*, **11**, 751–760.

40. Tompa, M., Li, N., Bailey, T. L., et al. (2005) Assessing computational tools for the discovery of transcription factor binding sites., *Nat. Biotechnol.*, **23**, 137–144.

41. Kuhn, D. E., Martin, M. M., Feldman, D. S., et al. (2008) Experimental validation of miRNA targets, *Methods*, **44**, 47–54.

42. VanGuilder, H. D., Vrana, K. E. and Freeman, W. M. (2008) Twenty-five years of quantitative PCR for gene expression analysis, *Biotechniques*, **44**, 619–626.

43. Alam, J. and Cook, J. L. (1990) Reporter genes: Application to the study of mammalian gene transcription, *Anal. Biochem.*, **188**, 245–254.

44. Prediger, E. A. (2001) Detection and quantitation of mRNAs using ribonuclease protection assays., *Methods Mol. Biol.*, **160**, 495–505.

45. Kurien, B. T. and Scofield, R. H. (2015) Western blotting: an introduction., *Methods Mol. Biol.*, **1312**, 17–30.

46. Bonner, W. A., Hulett, H. R., Sweet, R. G., et al. (1972) Fluorescence activated cell sorting., *Rev. Sci. Instrum.*, **43**, 404–9.

47. Manis, J. P. (2007) Knock Out, Knock In, Knock Down — Genetically Manipulated Mice and the Nobel Prize, *N. Engl. J. Med.*, **357**, 2426–2429.

48. Tiscornia, G., Singer, O., Ikawa, M., et al. (2003) A general method for gene knockdown in mice by using lentiviral vectors expressing small interfering RNA., *Proc. Natl. Acad. Sci. U. S. A.*, **100**, 1844–1848.

49. Shoemaker, B. a. and Panchenko, A. R. (2007) Deciphering protein-protein interactions. Part I. Experimental techniques and databases, *PLoS Comput. Biol.*, **3**, 0337–0344.

50. MacLachlan, T. K., Dash, B. C., Dicker, D. T., et al. (2000) Repression of BRCA1 through a feedback loop involving p53., *J. Biol. Chem.*, **275**, 31869–75.

51. Yang, S.-Z., Lin, F.-T. and Lin, W.-C. (2008) MCPH1/BRIT1 cooperates with E2F1 in the activation of checkpoint, DNA repair and apoptosis., *EMBO Rep.*, **9**, 907–15.

52. Chen, C. F., Li, S., Chen, Y., et al. (1996) The nuclear localization sequences of the BRCA1 protein interact with the importin-alpha subunit of the nuclear transport signal receptor., *J. Biol. Chem.*, **271**, 32863–8.

53. Zhang, J., Willers, H., Feng, Z., et al. (2004) Chk2 phosphorylation of BRCA1 regulates DNA double-strand break repair., *Mol. Cell. Biol.*, **24**, 708–18.

54. Xia, Y., Pao, G. M., Chen, H.-W., et al. (2003) Enhancement of BRCA1 E3 ubiquitin ligase activity through direct interaction with the BARD1 protein., *J. Biol. Chem.*, **278**, 5255–63.
